# Effect of wall thickness of 3D‐printed models on resisting deformation from thermal forming in‐office aligners

**DOI:** 10.1002/cre2.827

**Published:** 2024-02-03

**Authors:** Gregory W. Bennett, Tia DiGiovanni

**Affiliations:** ^1^ Department of Adult Restorative Dentistry University of Nebraska Medical Center College of Dentistry Lincoln Nebraska USA; ^2^ Fourth‐Year Dental Student University of Nebraska Medical Center College of Dentistry Lincoln Nebraska USA

**Keywords:** printing, three‐dimensional, Orthodontic appliances, removable, stereolithography

## Abstract

**Background:**

Fabricating clear aligners by thermoforming three‐dimensional printed dental models requires a high degree of accuracy. It is unknown whether model thickness affects the accuracy when used to thermoform aligners.

**Purpose:**

This research utilizes three‐dimensional printed models made with differing wall thicknesses to determine its effect on their ability to withstand deformation during aligner fabrication.

**Methods:**

A total of 50 models of different wall thickness (10 each of 0.5, 1.0, 1.5, 2.0 mm, and solid) were printed using model resin (Model V2, Formlabs) on a low‐force stereolithography printer (Form 3B, Formlabs). Aligners were then fabricated using a thermal pressure forming machine (Biostar V, Great Lakes Dental Technologies) utilizing 25 s cycles to adapt 0.030″ acrylic sheets (Invisacryl, Great Lakes Dental Technologies), then removed from the models and sprayed with a contrast powder (Optispray, Dentsply Sirona) to aid in scanning with an intraoral scanner (CEREC Primescan, Dentsply Sirona). Each aligner's data was then compared to the original file used for printing with 3D comparison software (Geomagic Control X, 3D Systems).

**Results:**

The results show model thickness greater than or equal to 2.0 mm produced clinically acceptable results within the margin of error (0.3 mm). A total of 0.5 mm thickness failed to withstand thermal forming in 4 of the 10 trials. A total of 0.5 mm produced 27.56% of results in tolerance, 1.0 mm produced 75.66% of results in tolerance, 1.5 mm had 80.38% of results in tolerance, 86.82% of 2 mm models were in tolerance, and solid had 96.45% of results in tolerance.

**Conclusion:**

Hollow models of thicknesses 2.0 mm and solid models produced clinically acceptable aligners while utilizing less resin per unit compared to solid models, thus being more cost effective, time efficient and eco‐friendly. Therefore, a recommendation can be made to print hollow models with a shell thickness of greater than 2.0 mm for aligner fabrication.

## 1 INTRODUCTION

The field of orthodontics has developed significantly throughout the years, with many advances in part due to digital dentistry. One common example is the shift away from physical impressions toward intra oral scanners, which patients find more comfortable (Christopoulou et al., 2022; Tarraf & Ali, 2018). The ability to scan a dental arch has led to several advancements, such as an increased availability of clear aligner therapy. Digital scans have proven to be able to capture the surface detail of a patient's mouth with a similar degree of accuracy (within a 0.10 mm overall deviation) as compared with alginate impressions (Lee & Park, 2020). The increase in accessibility and improvements in technology has led to the number of patients’ receiving clear aligner therapy increasing annually (Research GV Clear Aligners Market Size, 2022). With this progression there has been experimentation with in‐office fabrication of clear aligners. In‐office fabrication of clear aligners comes with significant economic benefits, but little is known about the most efficient and effective production methods for models (Tozlu & Ozdemir, 2021).

Varying stages in the digital workflow to fabricate clear aligners has been studied to see the impact on accuracy of the final product. The type of aligner material utilized (Nasef et al., 2017), the printer utilized (Groth et al., 2014), the print layer heights (Ledingham et al., 2016), the type of resin utilized (Kasper, 2020), layout of the build platform (Short et al., 2018), type of wash (Hwangbo et al., 2021), cure (Bayarsaikhan et al., 2021; McCarty et al., 2020), and drying conditions (Sherman et al., 2020) are among the most studied steps. Each of these studies has advanced the knowledge of how to accurately fabricate 3D printed items.

It is important to note that models are deemed clinically acceptable under the parameters they plan on being utilized for, and the same level of accuracy may not be needed for orthodontic purposes, in comparison to prosthodontic applications (Etemad‐Shahidi et al., 2020). Nonetheless, a high degree of accuracy is necessary in printed models to fabricate effective aligners and the understanding of the best techniques are continuously being adapted (Sabbagh et al., 2022).

To improve the print time and reduce cost and wanted materials, models can be 3D printed with a hollow geometry of varying wall thickness in comparison to the traditional solid fabrication. Although there are many positive associations with printing hollow models, the models still need to be strong enough to have the ability to withstand the thermoforming step to produce clinically acceptable aligners (Kasper, 2020).

While many of the studies have focused on refining and finding the best way to 3D print models for thermoforming aligners, innovation is far from static. A recent development is the introduction of Tera Herz TC‐85 resin by Graphy, which allows for the direct three‐dimensional printing of aligners, eliminating the need for physical models for thermoforming (Panayi, 2023). This new wave of advancements, with more companies following to develop this type of resin, has the potential for faster, more precise, and potentially more cost‐effective orthodontic care.

## 2 MATERIALS AND METHODS

**Figure 1:**
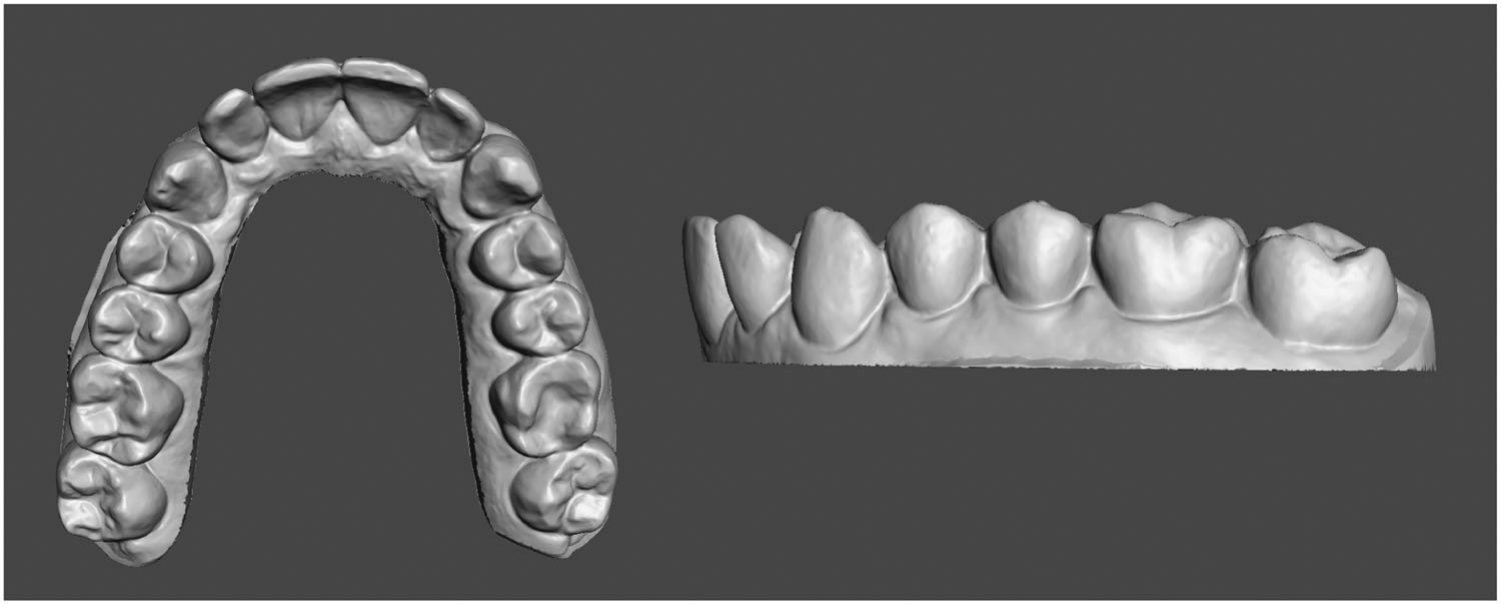
Solid 3D model of the maxillary arch.

**Figure 2:**
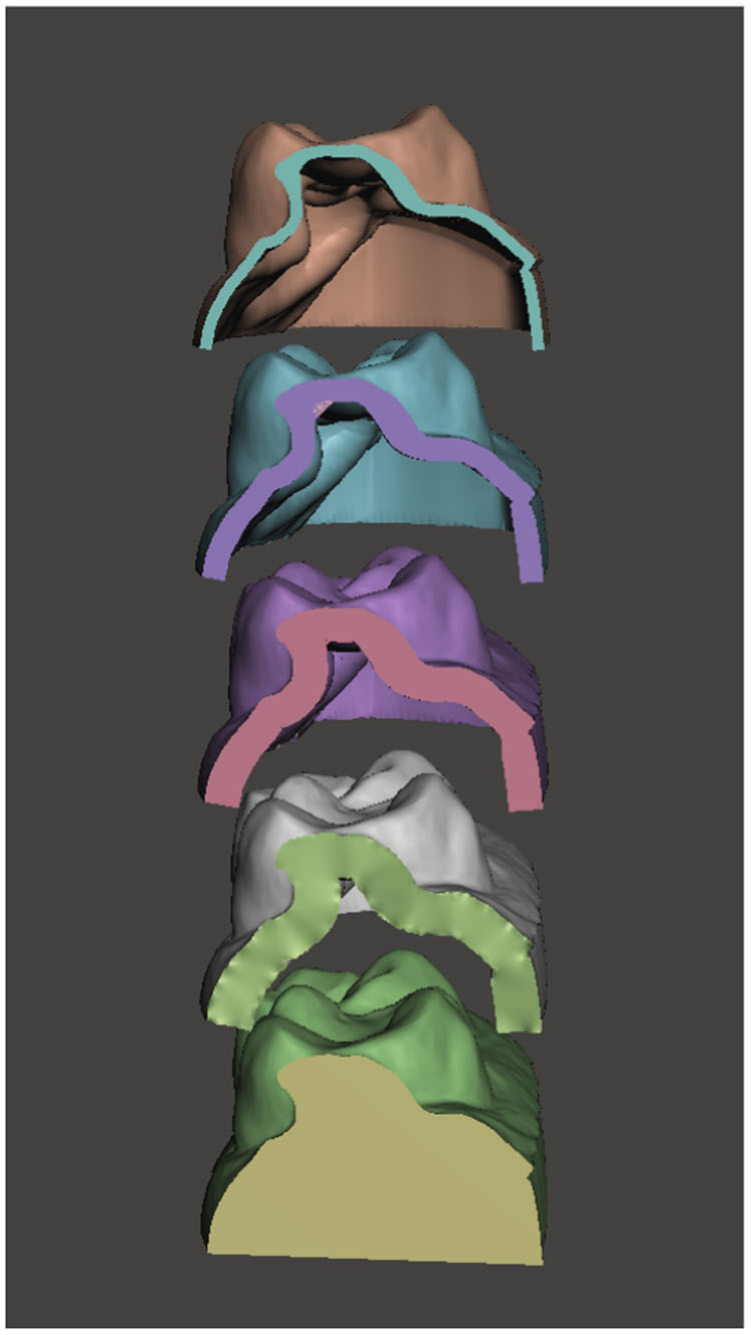
Cross section through 3D rendering showing in descending order: 0.5, 1.0, 1.5, 2.0 mm and solid models.

**Figure 3:**
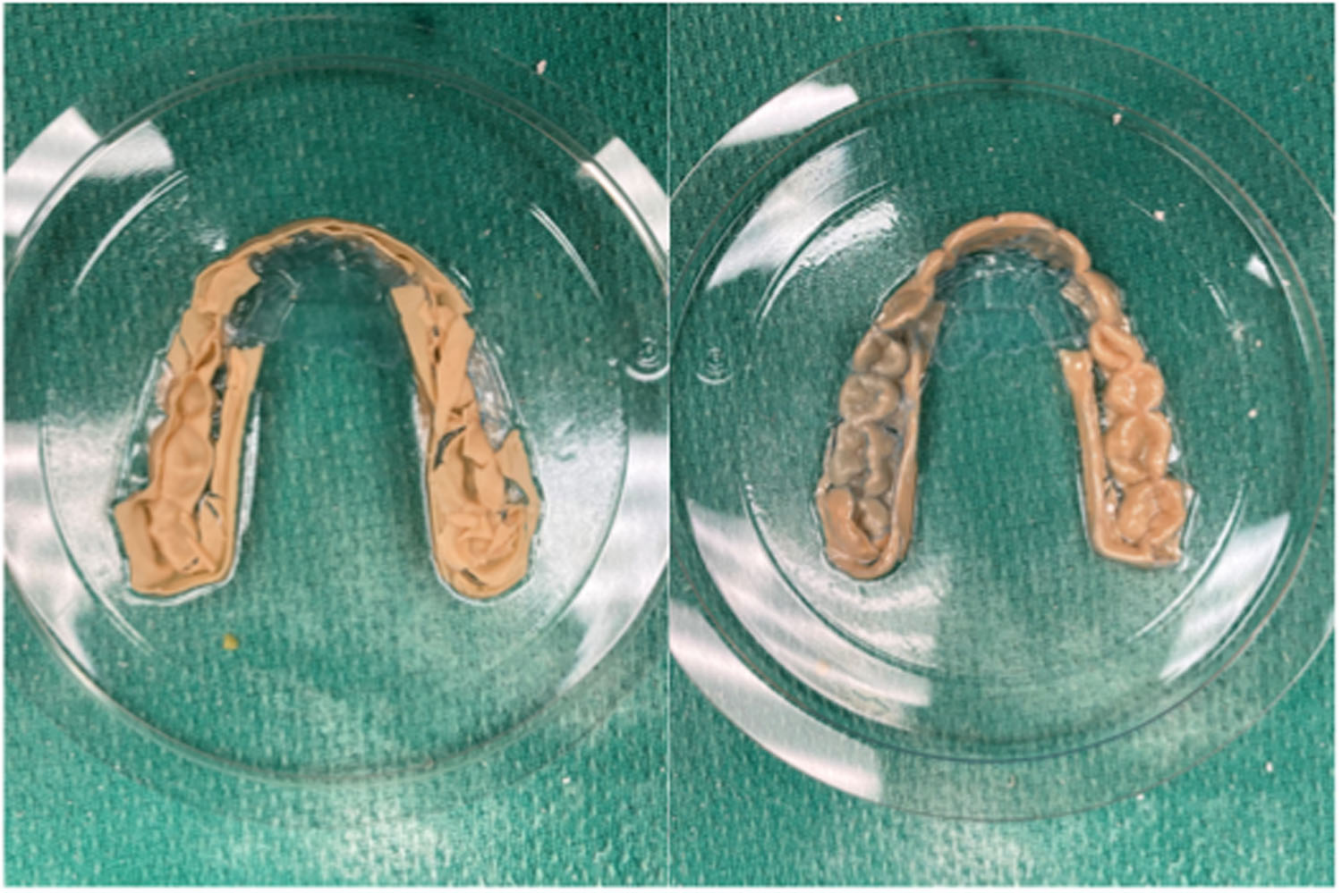
A model of 0.5 mm that did not survive pressure forming of the aligner.

**Figure 4:**
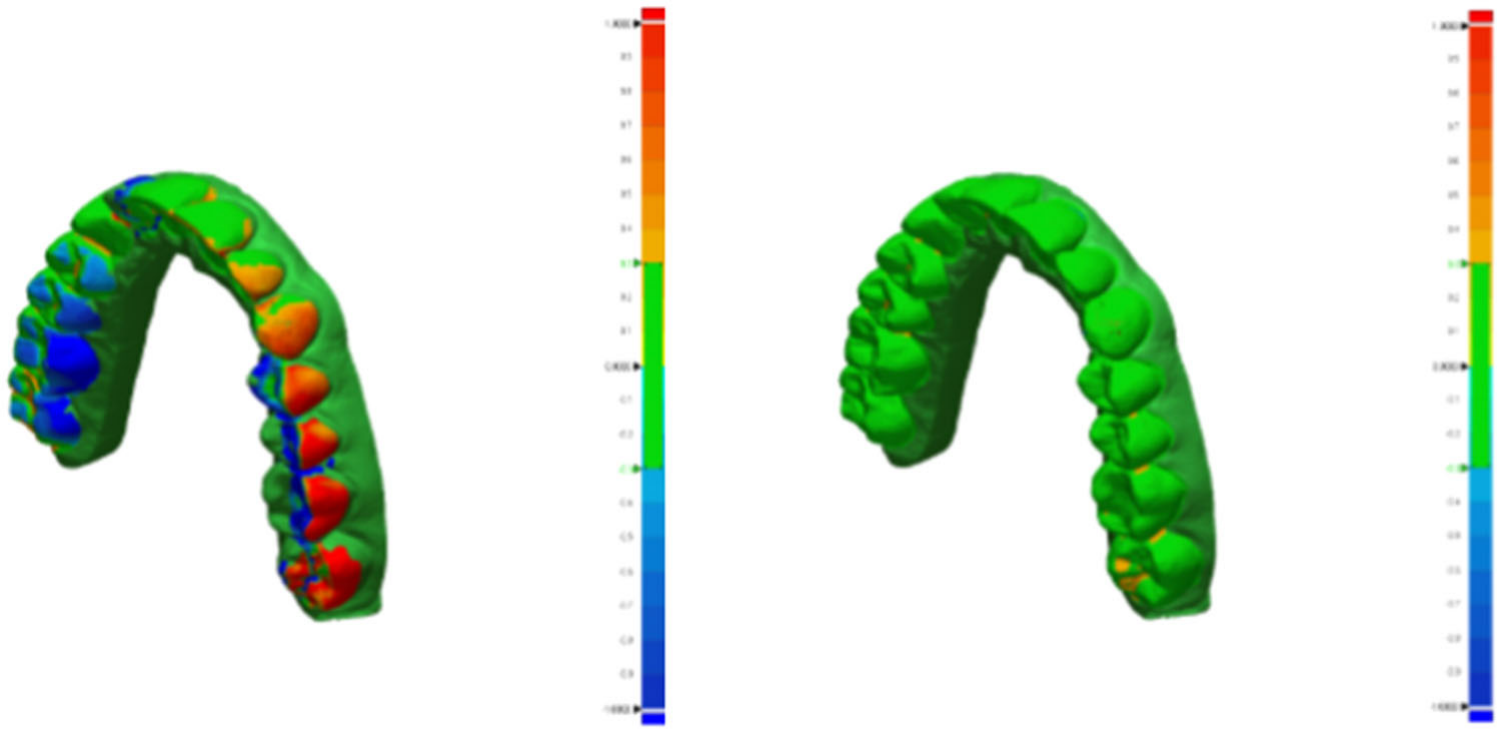
Color dimensional deviation maps were generated for each scanned aligner. The image is interior surface of the aligner overlayed onto the original 3D model. The dark blue color indicates negative deviations greater than 0.3 mm, and green areas indicate deviations less than 0.3 mm, which lie within the limits of clinical acceptability. A 0.5 mm model on the left shows significant deviation across the posterior dentition. A solid model on the right shows high accuracy with near complete green.

The maxillary arch of a patient was scanned with an intraoral scanner (Trios 3, 3Shape). The STL file was then converted into a 3D model capable of being printed using design software (Figure 1) (MeshMixer 3.2; Autodesk). This solid model was converted into hollow models of four varying thicknesses: 0.5, 1, 1.5, and 2 mm (Figure 2). The models were oriented for printing with the occlusal plane away from the print bed. Support structures were auto generated using the printer's software (Preform, Formlabs). Fifty models were printed in total, 10 of each wall thickness using model resin (Model V2, Formlabs) on a low‐force stereolithography printer (Form 3B, Formlabs). Flush cutters were then used to remove supports from the models. The models were then washed using 96% isopropyl alcohol with agitation for 10 min, followed by postprint cure for 30 min at 60°C (Form Cure; Formlabs). Each model was then sprayed with a releasing agent (Trim‐Rite, Dentsply Sirona) to assist aligner release from the printed model. Aligners were then fabricated using a thermal pressure forming machine (Biostar V, Great Lakes Dental Technologies) utilizing 25 s cycles at 87 psi to adapt 0.030″ acrylic sheets (Invisacryl, Great Lakes Dental Technologies). Models that experienced catastrophic compression during thermoforming were eliminated from analysis (Figure 3). Aligners were then removed from models, trimmed, and sprayed with a contrast powder (Optispray, Dentsply Sirona) to aid in scanning the intaglio surface with an intra oral scanner (CEREC Primescan, Dentsply Sirona). Each scanned aligner file was then digitally trimmed to the dentition (Meshmixer, Autodesk). The file was then compared to the original used for printing with 3D metrology comparison software (Geomagic Control X, 3D Systems). The software creates a deviation value for every vertex in the data against a predefined gap distance of 0.3 mm yielding the percent in tolerance values for each aligner (Systems, 2020). Color Deviation maps were exported that showed positive deviation along the surface greater than 0.3 mm as red and negative deviation greater than 0.3 mm as blue (Figure 4).

Statistical software (SPSS V29.0, IBM Corp) was used to conduct statistical analysis. The data failed the test for homogeneity of variance, therefore a Kruskal–Wallis test was performed. The null hypothesis that there would be no difference in median percent in tolerance between the sample groups was rejected *H*(4) = 29.73 *p* < .001 (Table 1). Pairwise comparisons by group were performed with a Bonferroni correction to identify significant differences between percent in tolerance across groups.

**Table 1:** The independent samples Kruskal–Wallis test summary shows significance with a *p* < .001.

Independent‐Samples Kruskal–Wallis test summary	
Total *N*	44
Test statistic	29.730^a^
Degree of freedom	4
Asymptotic Sig. (two‐sided test)	<0.001

^a^ The test statistic is adjusted for ties.

## 3 RESULTS

**Graph 1:**
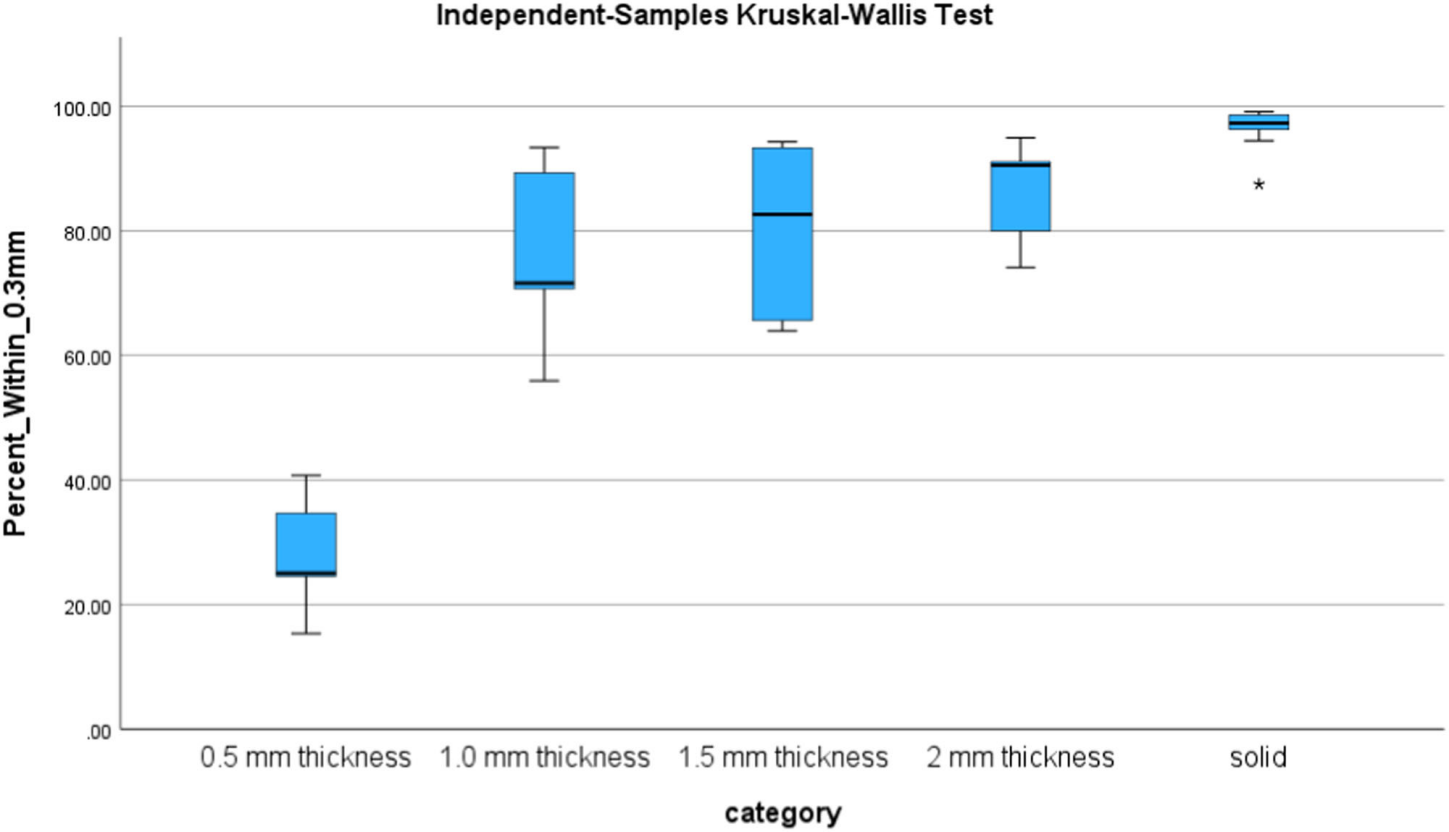
The greatest deviations between models after thermo‐forming were evident in the 0.5 mm group, demonstrating only 27.57% of data points in tolerance. In contrast, all other thicknesses demonstrated more than 75% of data points in tolerance.

A total of 44 of the original 50 models were able to be analyzed. Several models with thinner walls did not survive thermoforming (Figure 3). A total of 6 models deformed during aligner fabrication, 4 of the 0.5 mm (40%) and 1 each of 1.0 (10%) and 1.5 mm (10%). The median percent in tolerance is demonstrated in Table 2, and it can be observed how it increased as the shell thickness increased. Both Table 2 and Graph 1 show the generalized increase in percent of data points within the 0.3 mm tolerance (27.56% for 0.5 mm, 75.66% for 1.0 mm, 80.38% for 1.5 mm, 86.82% for 2 mm, and 96.45% for solid). This can visually be observed by looking at Figure 4 where color dimensional deviation maps are shown. The dark blue color indicates negative deviations greater than 0.3 mm, and green areas indicate deviations less than 0.3 mm, which lie within the limits of clinical acceptability. A 0.5 mm model on the left shows significant deviation across the posterior dentition. A solid model on the right shows high accuracy with near complete green. The Pairwise comparisons with Bonferroni correction show significant differences between group comparisons. Table 3 shows the statistically significant between group comparisons. The 0.5 mm group was significantly different from 2.0 mm (*p* = .01), and solid (*p* < .01). The 1.0 mm group was significantly different from the solid (*p* < .01). The 1.5 mm group was significantly different from solid (*p* < .01) the 2.0 mm group was not significantly different from the solid group (*p* = .22).

**Table 2:** This descriptive statistics table demonstrates the mean percent in tolerance of each group studied as well as the success frequency of each thickness (N).

Descriptives								
Percent_within_0.3 mm	*N*	Mean	Standard deviation	Standard error	95% Confidence interval for mean		Minimum	Maximum
					Lower bound	Upper bound		
0.5 mm thickness	6	27.5673	8.89703	3.63220	18.2304	36.9041	15.35	40.74
1.0 mm thickness	9	75.6866	13.34001	4.44667	65.4326	85.9407	55.89	93.38
1.5 mm thickness	9	80.3853	12.86946	4.28982	70.4929	90.2776	63.90	94.30
2 mm thickness	10	86.8232	7.77813	2.45966	81.2591	92.3874	74.06	94.98
solid	10	96.4595	3.44980	1.09092	93.9916	98.9273	87.52	99.12
Total	44	77.3381	23.28933	3.51100	70.2575	84.4187	15.35	99.12

**Table 3:** Pairwise comparison shows significant differences between group comparisons.

Pairwise comparisons of category					
Sample 1–Sample 2	Test statistic	Standard error	Standard test statistic	Sig.	Adj. Sig.^a^
0.5 mm thickness–1.0 mm thickness	−13.722	6.768	−2.028	0.043	0.426
0.5 mm thickness–1.5 mm thickness	−17.167	6.768	−2.536	0.011	0.112
0.5 mm thickness–2 mm thickness	−21.300	6.631	−3.212	0.001	0.013
0.5 mm thickness–solid	−34.500	6.631	−5.203	<0.001	0.000
1.0 mm thickness–1.5 mm thickness	−3.444	6.053	−0.569	0.569	1.000
1.0 mm thickness–2 mm thickness	−7.578	5.900	−1.284	0.199	1.000
1.0 mm thickness–solid	−20.778	5.900	−3.522	<0.001	0.004
1.5 mm thickness–2 mm thickness	−4.133	5.900	−0.701	0.484	1.000
1.5 mm thickness–solid	−17.333	5.900	−2.938	0.003	0.033
2 mm thickness–solid	−13.200	5.743	−2.299	0.022	0.215

*Note*: Each row tests the null hypothesis that the Sample 1 and Sample 2 distributions are the same. Asymptotic significances (two‐sided tests) are displayed. The significance level is 0.050.

^a^ Significance values have been adjusted by the Bonferroni correction for multiple tests.

## 4 DISCUSSIONS

The goal of this study was to determine if 3D printing hollow models in comparison to solid models will have an impact on the accuracy of thermoformed in office clear aligners and to determine the minimum shell thickness necessary to produce clinically acceptable aligners capable of withstanding thermoforming. The results of this study showed that significant deformations occurred for models printed with less than 2.0 mm of thickness. This allowed us to reject the null hypothesis that all model thicknesses will produce the same amount of dimensional accuracy when thermoformed for orthodontic appliances.

Currently there is yet to be a clear, evidence‐based numerical value to use for percent of data points in tolerance to deem clinically acceptable. When determining what value to use, past studies were evaluated, which kept clinical applications of clear aligners in mind. Several different values of percent in tolerance have been proposed in the literature ranging from 0.03 to 0.3mm (Wesemann et al., 2017; Zhang et al., 2019). Kenning chose 0.25 mm as percent in tolerance by evaluating Align technology to understand that deviations must be less than 0.25–0.3 mm for teeth to move orthodontically (Kenning et al., 2021). With this same justification we choose 0.3 mm as our accepted tolerance threshold for the study. Geomagic comparison software produced color deviation maps where green showed data points in tolerance (−0.3 mm < X < +0.3 mm) and gave data points under tolerance (X < −0.3 mm) in blue and over tolerance (X < + 0.3 mm) in red. Statistical significance was determined based on the percentage of data points that fell within tolerance, as any point falling outside the 0.3 mm tolerance would produce aligners that are ill fitting.

When observing both the models and the aligners fabricated from 0.5 mm models, it was visibly apparent that it would not fit on the patient's dentition. Not only were some of the 0.5 mm models unable to be removed from their supports without fracturing, but those who did underwent significant plastic deformation. Some plastic deformation was also noted on the 1.0 mm models after thermoforming aligners, where it was apparent that even if they did fit onto the patient's dentition, they would produce unwanted orthodontic movements.

To date, there is only one study that evaluated the dimensional accuracy of appliance fabricated on hollow models. This study aimed to evaluate the effect of wall thickness of 3D printed models on the accuracy of pressure formed clear aligners, which produced results that supported our results of 2.0 mm thickness being the minimum thickness able to produce clinically acceptable aligners (Kim et al., 2018). The data from the study show that hollow models of thickness 2.0 mm produce clinically acceptable aligners while utilizing less resin per unit compared to solid models. For the model studied, there was a 44% reduction in material usage when comparing the 2.0 mm model to the solid model. Not only does this significantly reduce unwanted waste but considering the current cost of resin for this printer at $149 per liter, there was also a reduction in cost from $1.06 for 2.0 mm thickness compared to $1.88 for solid models.

A recent study comparing the accuracy of orthodontic models printed using 4 different types of 3D printers found that stereolithography technology produced orthodontic models with greater accuracy than DLP or LCD technology (Grassia et al., 2023).

Limitations to this study include sources of error observed, such as during scanning of the aligners. While it would have been ideal to utilize the lab desktop scanner, the inability of the scanner to recognize the clear aligners, even with the addition of contrast spray, made it not feasible. Because of this, an intraoral scanner was used. The flexibility of aligners also led to difficulty with scanning as it is possible that aligners flexed during scanning. In addition, the contrast powder that was sprayed on the aligners to reduce opacity added a thickness that was not calculated into the results. Studies found that the added thickness added ranged from 0.0189 to 0.09 mm (Edelmann et al., 2020; Kim et al., 2018; Lehmann et al., 2011).

While printing hollow is an improvement that decreases the amount of wasted materials and decreases print time, more studies are evaluating the ability to completely bypass the need for a physical version of orthodontic models while fabricating aligners. It can be postulated that the future of 3D printing will allow a complete elimination of the need for clear aligners to be fabricated of dental models. A new resin, called TC‐85DAC (Graphy), was the initial directly printed aligner fabricated, with more companies continuing to release aligner resins as well (Panayi, 2023).

Much of the research and knowledge of direct 3D printed aligners is in the early stage. An early study by Can looked at TC‐85DAC resin resistance to wear after a 1‐week period, and found that they were not affected after a 1‐week service period (Can et al., 2022). In a review on the current state and future possibilities Tartaglia presented many concerns or unknowns regarding direct printing of aligners, including, cytotoxicity, varying thickness after printing (Tartaglia et al., 2021) More recent studies evaluating the biocompatibility of a resin formulated for direct printed aligners (TC85A, Graphy) and found high biocompatibility at 14 days (Pratsinis et al., 2022) and when postprocessed with the manufacturers curing unit (Alessandra et al., 2023) (THC2, Graphy).

One such study by Edelmann evaluated the ability to directly print aligners and found that there was an increase in aligner thickness by approximately 0.2 mm. Additionally, Cole found that although traditional vacuum‐formed retainers showed the least amount of deviation and 3D printed models showed the greatest deviation, they all fell within 0.5 mm of the original model (Cole et al., 2019). However, materials are currently available to directly print thicker appliances, such as retainers and mouth guards, thus providing some hope that the fabrication of aligners in a similar manner may not be far behind (Nasef et al., 2017). Because materials to directly print aligners are in their infancy and may not be reliable to use today, it is important to utilize other knowledge to reduce waste of printing materials, such as printing models with a hollow shell. With this information, the limitations of direct printing of aligners can be appreciated which have the potential to affect the clinical ability to move teeth in a precise manner.

## 5 CONCLUSION

The accuracy of thermoformed aligners fabricated from 3D printed dental models is affected by the shell thickness of the models. If a shell thickness is printed too thin, it will not be able to withstand printing and/or thermoforming, thus the aligners fabricated will be inaccurate and ineffective, producing unwanted orthodontic movements. A recommendation to clinicians printing in‐office aligners can be made to print hollow models with a shell thickness of 2.0 mm to improve time efficiency of printing the models, reduce the cost of models, and minimize materials wasted, thus being more eco‐friendly.

## AUTHOR CONTRIBUTIONS

**Gregory Bennett**: Conceptualization (lead); Methodology (lead); formal analysis (lead); writing—review and editing (lead). **Tia DiGiovanni**: writing—original draft (lead); Investigation (lead); writing—review and editing (supporting).

## CONFLICT OF INTEREST STATEMENT

The authors declare no conflicts of interest.

## Data Availability

Data available on request from the authors.
